# Leptospirosis? An epidemiologic investigation following the historic 2024 floods in Rio Grande do Sul, Brazil

**DOI:** 10.1016/j.onehlt.2025.101146

**Published:** 2025-07-19

**Authors:** Christopher J. Hernandez, Greici Gunzel, Clarice Ritter, Roberto Carlos Freitas Bugs, Thiago Rocha, Trevon Fuller, Patricia Brasil, Ivana Rosângela dos Santos Varella, Maria da Graça Pimenta Machado, Carina Guedes Ramos, Ângela Piccoli Ziegler, Breno Riegel Santos, Marineide Gonçalves de Melo, Karin Nielsen-Saines

**Affiliations:** a UCLA David Geffen School of Medicine, Los Angeles, CA, USA; b Department of Infectious Diseases, Hospital Nossa Senhora da Conceição, Sistema Único de Saúde, Porto Alegre, Brazil; c Instituto Nacional de Infectologia Evandro Chagas, Fiocruz, Rio de Janeiro, Brazil; d Department of Epidemiology and Public Health, Hospital Nossa Senhora da Conceição, Sistema Único de Saúde, Porto Alegre, Brazil; e Department of Pediatrics, Division of Pediatric Infectious Diseases, UCLA David Geffen School of Medicine, Los Angeles, CA, USA

**Keywords:** Outbreak investigation, Vector-borne, Zoonotic, Leptospirosis, Dengue, Flood, South Brazil

## Abstract

In May 2024, the Brazilian state of Rio Grande do Sul experienced an historic flood that affected millions of people. Hundreds of thousands of people were displaced by the record-breaking flood waters, exposing them to water-borne pathogens, including leptospirosis. With concern for an outbreak, local efforts focused on identifying and treating any potential cases of leptospirosis. Using data from a local database for people presenting with signs and symptoms compatible with leptospirosis, we conducted a retrospective-prospective cohort study to estimate the incidence of confirmed cases. Of 539 patients reported to the database, a total of 485 patients were tested for leptospirosis and 303 were tested for Dengue (with 283 testing for both pathogens). We found 17 (3.5 %) confirmed cases of leptospirosis and 102 (33.7 %) confirmed cases of dengue fever. Most tested negative for both, with several confirmed cases of Hantavirus, Influenza A, and HIV. Although there were no significant differences between diagnoses and exposure risk factors, we did find several clinical differences, including headache, respiratory symptoms, diarrhea, acute kidney injury, jaundice, bleeding diathesis, and pulmonary hemorrhage. Leptospirosis was also found to be significantly associated with adverse clinical outcomes when compared to both dengue and the Unrelated/Unknown illness group and more likely to be of the male sex. These data reinforce the need for broader systematic surveillance for pathogens that may circulate in the event of major environmental disasters. Further, stronger diagnostics are urgently needed to distinguish between the causes of largely febrile outbreak in the face of a changing climate.

## Highlights

• Large study of people with documented exposures to contaminated water and land following historic floods.
• First study assessing the true prevalence of suspected Leptospirosis cases in south Brazil.
• Dengue was found to be prevalent in an unexpected region of Brazil.
• Evidence that other pathogens, such as Hantavirus and Influenza, were circulating as well.
• We highlight the need for rethinking outbreak investigations in environments affected by climate change.

Leptospirosis, caused by bacterial spirochetes belonging to pathogenic *Leptospira* spp.*,* is a common zoonotic disease worldwide [1], ranging in clinical severity from a non-specific fever to Weil's disease characterized by fever, renal failure, jaundice, hemorrhage, and respiratory distress [1]*.* The bacterium is normally found in rodent and livestock reservoirs and is excreted through urine with the ability to persist in contaminated water or soil for months under favorable environmental conditions [2]. Zoonotic transmission occurs when people come into direct contact with contaminated water or soil [1].

In the event of flooding, dispersal of *Leptospira* spp. can lead to large disease outbreaks [3]. Well documented leptospirosis epidemics following flooding events have occurred in Malaysia, Greece, India, and China [[4], [5], [6], [7]]. Leptospirosis outbreaks have also been documented in Brazil [8,9]. Between 2010 and 2019, the state of Rio Grande do Sul had 4760 confirmed cases and 238 deaths with an estimated 5 cases per 100,000 inhabitants compared to the national Brazilian average of 1.9 cases per 100,000 [10]. Rio Grande do Sul has a large urban non-native Brown rat (*Rattus norvegicus*) population, a dominant rural livestock industry, and climatic factors that favor environmental dispersal and persistence, which may explain the disproportionate incidence of leptospirosis compared to the rest of Brazil [11].

In May 2024, the state of Rio Grande do Sul experienced its worst flooding event in over 80 years [12]. It surpassed all historical records, with the Guaíba river reaching 5.31 m (17.4 ft) [13]. The recent flooding event impacted a population of 2.4 million, displaced 500,000 individuals, and killed 183 people [14]. Nearly 78,000 residents required rescue from floodwater [14]. Following the floods, there was a high concern for an outbreak of leptospirosis across the state. On May 5, 2024, the Health Surveillance Directorate recommended antibiotic therapy for patients with symptoms and risk factors compatible with leptospirosis [15]. By June, the Municipal Health Department of Porto Alegre received 1756 reports of suspected cases of leptospirosis, with 1418 still under investigation at that time [16].

The vector-borne flavivirus, *Dengue virus* (DENV), commonly co-occurs with cases of leptospirosis as floodwater creates breeding grounds for the mosquito vector, *Aedes aegypti* [17]. Further, dengue fever (DF) and leptospirosis are difficult to distinguish clinically, especially in the early stages of disease [[17], [18], [19]]. Co-occurring outbreaks can complicate the understanding of an epidemic if the precise diagnoses are not established. To reliably distinguish them, direct laboratory testing through Polymerase Chain Reaction (PCR), antigenemia tests, or serology measurements are required [20].

Using data from a database of 539 suspected cases of leptospirosis, we aimed to estimate the true prevalence of leptospirosis, characterize co-occurring epidemics, identify epidemic risk factors, map the geographic distribution of cases, and report on pertinent disease-specific clinical and laboratory data.

## 1 Methods

This was a prospective-retrospective cohort study which evaluated medical records of patients reported to the Brazilian Single Unified System (SUS) database as suspected leptospirosis cases between May 4th, 2024, to September 30th, 2024, following the floods in Porto Alegre, Brazil. Patients reported to this database first presented to outpatient clinics across the city or were hospitalized due to signs and symptoms suggestive of leptospirosis infection. At their initial encounter with the health system, serologic and molecular diagnostic testing was performed by two reference laboratories. At the Laboratório Central do Estado do Rio Grande do Sul (Lacen-RS), DENV infection was evaluated using two enzyme-linked immunosorbent assays (ELISA): the Dengue IgM Capture ELISA kit (Panbio Inc., Columbia, MD, USA) for detection of IgM antibodies, and the Early ELISA NS1 kit (Panbio Inc., Columbia, MD, USA) for detection of NS1 antigen. Leptospirosis was assessed via ELISA using the Panbio Leptospira IgM ELISA kit (Panbio Inc., Columbia, MD, USA) if symptom onset was more than 7 days, or by PCR if duration of symptoms was 7 days. Patients in the initial database were invited to return to the infectious diseases clinic of the Instituto de Pesquisa Clinica do Rio Grande do Sul (IPARGS) affiliated with the Hospital Nossa Senhora da Conceição where IgM and IgG serologies specific for both leptospirosis and DENV were measured.

To determine demographic and clinical differences between leptospirosis and DENV infections, we included only patients who had both infections assessed at the initial health system encounter and/or during follow-up. Patients who tested for both leptospirosis and DENV at least once and had negative results for both were classified as having Unrelated/Unknown Illness (UI). Because of the lack of systematic surveillance for pathogens besides DENV and leptospirosis, we included other confirmed infections in the UI group.

Data was extracted from medical records including sociodemographic characteristics. Age was kept as a continuous variable. Race or ethnicity was categorized according to hospital records: White, Black, or Parda (mixed race). Education was categorized as ≥8 years or < 8 years of schooling. Admission to the intensive care unit (ICU) and mortality events were determined by medical records.

All patients were asked a standard set of questions used by the Brazilian Ministry of Health to examine signs and symptoms as well as the type of exposures that placed them at risk [21]. The information was collected by healthcare providers.

### 1.1 Statistical analysis

Descriptive statistics were used to summarize demographic characteristics and clinical information. Demographics of patients who underwent both DENV and leptospirosis testing were described to ensure no bias. The demographics, risk factors, signs and symptoms, and clinical characteristics of patients between diagnoses were compared. Bivariate two-tailed chi-square tests, when *n* ≥ 5, and Fisher's exact tests, when *n* < 5, were used to determine statistical significance in sociodemographic factors. Fisher's exact test was used to determine differences in signs and symptoms. The median and interquartile range (IQR) were calculated for all laboratory values. Kruskal-Wallis tests were conducted to assess global differences. Pairwise Mann-Whitney *U* tests were performed to determine specific differences.

To examine differences in demographics and adverse outcomes between diagnoses, three multinomial logistic regression models were built, including age, sex, and race. Education was also explored to examine the impact of risk mitigation. Adverse outcomes were included to determine association of diagnosis with severity. In the first two models, UI was set as the base for comparison, while in the third model, DF was set as the base. Models were checked for collinearity using Variance Inflation Factor. Model fitness was verified by the Pseudo R-squared result. Corresponding subjects exhibiting missing data points for certain variables were not included in each corresponding analysis. All analyses were conducted using STATA version 14 (College Station, TX).

Ethics Statement: The study was approved by the Conceição Hospital Institutional Review Board (IRB). Written informed consent was obtained from all study participants who took part in the prospective arm of the study.

## 2 Results

**Fig. 1:**
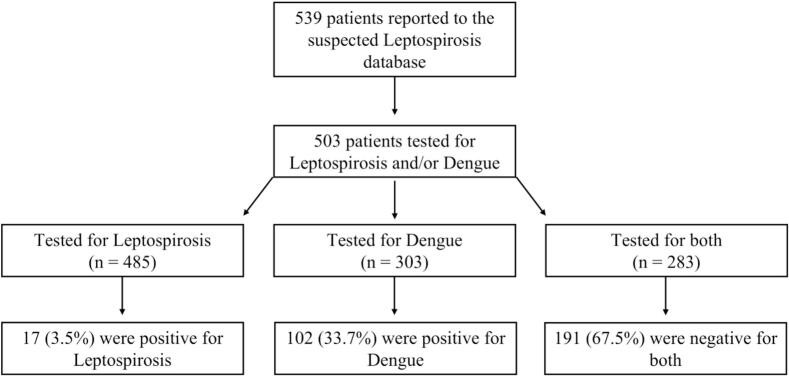
For study inclusion, participants had to test positive for leptospirosis, DENV, or test negative for both pathogens (assigned to the Unknown/Unrelated illness category) at the first visits and/or the follow-up visit. 202 among the 485 who tested for leptospirosis did not test for DENV. 20 among the 303 who tested for DENV did not test for leptospirosis. Of the 191 patients who tested negative for both DENV and leptospirosis, we identified 2 influenza A cases, 2 Hantavirus cases, 2 cases of advanced malignancy with associated sepsis, and 1 acute HIV infection and were included in the UI group.

In total, 539 patients were reported to the suspected leptospirosis database (Fig. 1). Of these, 485 had testing for leptospirosis infection performed: 376 had leptospiral DNA PCR tests, 92 had leptospiral-specific IgM serologies, and 8 had both assessments. An additional 9 had convalescent IgM/IgG testing performed on follow-up. For DENV testing, 261 had antigen EIA tests and 42 had DENV screening with serologic tests performed on follow-up for a total of 303 tested patients. In total, 283 patients had testing for both leptospirosis and DENV. Demographic data was similar between those who tested for leptospirosis, those who tested for DENV, and those who tested for both (Table S1). Five hundred and three participants were tested for leptospirosis and/or DENV during the first visit, and 97 returned for a follow-up visit. For patients who returned for a follow-up visit, the mean interval between visits was 90 days (range: 52 to 162 days). In total, 17 (3.5 %) patients tested positive for leptospirosis, all during the first visit. Among 102 (33.7 %) patients who tested positive for DF, 93 were diagnosed during the first visit, 7 were diagnosed in the follow-up visit, and 3 tested negative at the first visit but tested positive upon follow-up. Among 283 participants who were tested for both pathogens, 191 (67.5 %) tested negative for both. Within the UI group, there were 7 confirmed diagnoses, including 2 *influenza A* cases, 2 Hantavirus cases, 2 cases of malignancy with sepsis, and 1 case of acute HIV infection. Potential pathogens associated with flooding events that could represent other UI are listed in Table 1.

**Table 1:** Infectious diseases commonly associated with floodwaters in Brazil and globally.

Transmission Route	Disease	Pathogen(s)	Clinical Syndrome	Incubation Period	Diagnostic Methods
**Waterborne (ingestion/contact)**	Leptospirosis a	Pathogenic Leptospira spp. *(L. interrogans, L. kirschneri, L. borgpetersenii)*	Fever, conjunctival suffusion, jaundice, AKI, myalgia, pulmonary hemorrhage	2–30 days (avg. 7–14)	Serology (MAT, ELISA), PCR, culture
	Hepatitis A / E	Hepatitis A/E viruses	Acute hepatitis: jaundice, anorexia, nausea	15–50 days (HAV), 15–60 (HEV)	Serology (IgM), PCR
	Typhoid / Paratyphoid fever	*Salmonella Typhi*, *S. paratyphi*	Prolonged fever, abdominal pain, diarrhea/constipation	6–30 days (avg. ∼10–14)	Blood/stool culture
	Bacterial gastroenteritis	*E. coli*, *Shigella*, *Campylobacter*, *Salmonella* spp.	Fever, abdominal pain, diarrhea (sometimes bloody)	1–7 days depending on pathogen	Stool culture, PCR
	Amebiasis / Giardiasis / Cryptosporidiosis	*E. histolytica*, *Giardia*, *Cryptosporidium*	Diarrhea, bloating, weight loss (chronic possible)	1–4 weeks (avg. 7–10 days)	Stool O&P, antigen, PCR
	Norovirus / Rotavirus	Norovirus, Rotavirus	Vomiting, diarrhea, fever	12–48 h (Norovirus); 1–3 days (Rotavirus)	PCR, ELISA
**Rodent exposure (post-flood cleaning, rural areas)**	Hantavirus Pulmonary Syndrome (HPS)	Hantavirus (e.g., Juquitiba, Araraquara genotypes)	Fever, myalgia, GI symptoms, rapid respiratory failure, shock	Serology (IgM/IgG), RT-PCR, clinical suspicion	1–5 weeks (typically 2–4 weeks)
**Aerosolized water (showers, stagnant tanks)**	Legionellosis (Legionnaires' Disease)	*Legionella pneumophila*	Atypical pneumonia, fever, cough, confusion	2–10 days (avg. 5–6)	Urine antigen, PCR, BCYE culture
**Vector-borne (mosquitoes breeding in stagnant water)**	Dengue	*Dengue virus*	Fever, rash, retro-orbital pain, myalgia, hemorrhagic signs	4–10 days	NS1 antigen, IgM/IgG, PCR
	Zika	*Zika virus*	Fever, rash, arthralgia, conjunctivitis	3–14 days	PCR (serum/urine), IgM
	Chikungunya	*Chikungunya virus*	Fever, severe joint pain, rash	2–12 days	PCR, IgM serology
	Yellow fever	*Yellow fever virus*	Fever, rash, myalgias, nausea, headaches, jaundice, multi organ failure	3–6 days	PCR, IgM serology
**Soil/mud exposure**	Tetanus	*Clostridium tetani*	Trismus, rigidity, spasms	3–21 days	Clinical diagnosis
	Cutaneous mycoses / Sporotrichosis	*Sporothrix schenckii*, dermatophytes	Ulcers, nodules, lymphangitis (sporotrichosis)	1–12 weeks	Fungal culture, biopsy
**Respiratory (overcrowding, mold exposure)**	Influenza, RSV, COVID-19	Respiratory viruses	Cough, fever, myalgia, respiratory distress	1–4 days (Influenza), 2–14 (COVID-19)	PCR, rapid tests
	Tuberculosis (reactivation)	*M. tuberculosis*	Cough, weight loss, fever, night sweats	Weeks to years (latent reactivation)	Sputum smear, culture, GeneXpert

a Leptospirosis can remain in the environment under favorable conditions and be associated with contaminated water, soil, mud, as well as contact with rodents or livestock.

The median participant age (interquartile range) was 38 years (26, 51) (Table 2). Approximately 61 % of patients were male. Among participants with leptospirosis, 88 % were male as compared to 51 % with DF, *p* = 0.004. Most patients (78.2 %) identified as white, 12.9 % identified as Black, and 8.9 % identified as mixed race. In total, 47.2 % reported less than 8 years of schooling. Most patients (83.5 %) received outpatient healthcare while 16.5 % were hospitalized. Most patients with leptospirosis (58.8 %) were hospitalized compared to 14.7 % of those with DF, *p* < 0.001. Similarly, 47.1 % of patients with leptospirosis were admitted to the intensive care unit (ICU) and 11.8 % died as compared to 2.0 % of patients with DF, *p* = 0.001 (Table 2).

**Table 2:** Demographic and clinical outcome comparisons between those who were positive for dengue fever, leptospirosis, or an unknown/unrelated illness (UI) (*n* = 303).

	All	Dengue fever (*n* = 102)	Leptospirosis (*n* = 17)	UI⁎ (*n* = 191)	P – value
Age (years) Median (IQR)	38 (26, 51)	38.5 (27, 53)	39 (27, 60)	40.5 (27, 52)	0.852^a^
Sex					
Male	186 (61.4)	52 (51.0)	15 (88.2)	119 (64.7)	0.004^b^
Female	117 (38.6)	50 (49.0)	2 (11.8)	65 (35.3)	
Race					
White	236 (78.2)	83 (81.4)	14 (87.5)	139 (75.5)	0.766^c^
Black	39 (12.9)	12 (11.8)	2 (12.5)	25 (13.6)	
Mixed	27 (8.9)	7 (6.8)	0 (0.0)	20 (10.8)	
Education					
≤ 8 years	141 (47.2)	46 (46.0)	11 (68.8)	84 (45.9)	0.233^c^
> 8 years	158 (52.8)	54 (54.0)	5 (31.3)	99 (54.1)	
Healthcare					
Outpatient	253 (83.5)	87 (85.3)	7 (41.2)	159 (86.4)	0.001^c^
Hospitalization	50 (16.5)	15 (14.7)	10 (58.8)	25 (13.6)	
Adverse outcome					
ICU admission	20 (39.2)	2 (2.0)	8 (47.1)	10 (5.2)	0.001^b^
Death	6 (2.0)	2 (2.0)	2 (11.8)	2 (1.1)	

a ANOVA was used to compare differences in ages between groups.

b Fischer's exact test was used when n < 5 for a given cell.

c Chi-square tests of independence were used when n ≥ 5 for all cells.

⁎ Includes 7 cases where pathogens where other etiologies were identified: 2 influenza A cases, 2 Hantavirus cases, 2 cases of advanced malignancy with associated sepsis, and 1 acute HIV infection.

**Fig. 2:**
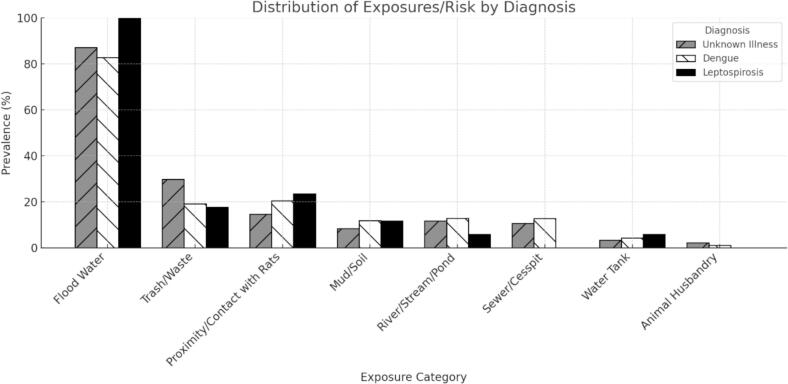
Distribution of exposure risk factors by final diagnosis of patients reported to the leptospirosis database.

The most reported risk factor was exposure to floodwater, affecting 86.5 % of participants, including 100 % of those diagnosed with leptospirosis (Fig. 2). Other frequently reported exposures included contact with trash (25.6 %), proximity to or contact with rats (17.0 %), and contact with a natural body of water (11.8 %). There were no significant differences in the types of risk exposures. Nearly all patients in this study resided in the Northern Metropolitan Region of Porto Alegre (Fig. S1).

**Fig. 3:**
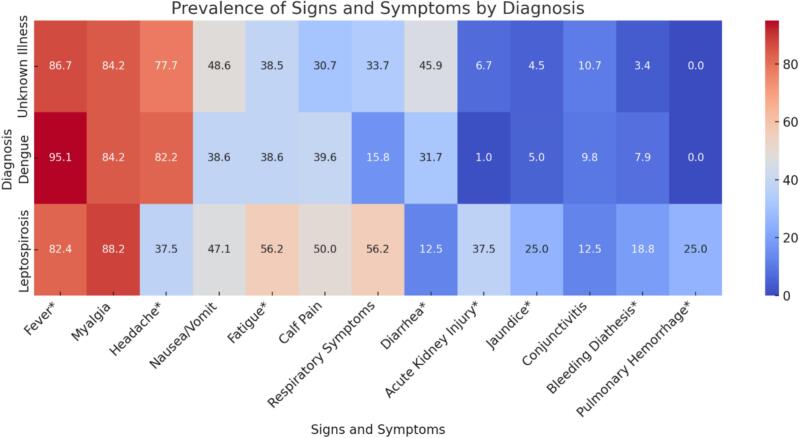
Heat map demonstrating the frequency of signs and symptoms described stratified by final diagnosis of patients reported to the leptospirosis database. * Fishers Exact test of significance was used to determine significant differences.

There were significantly different frequencies of specific signs and symptoms across the three diagnostic groups, including headache, respiratory symptoms, diarrhea, acute kidney injury (AKI), jaundice, bleeding diathesis, and pulmonary hemorrhage (Fig. 3) Fever was the most common symptom, reported in 89.3 % of patients overall, followed by headache (77.1 %), diarrhea (39.3 %), and respiratory symptoms (28.9 %). The most striking differences were seen in AKI, observed in only 6.4 % of cases overall but in 37.5 % of leptospirosis cases. Jaundice was observed in 5.8 % but 25.0 % of those with leptospirosis. Bleeding diathesis was observed in 5.7 % of patients but in 18.8 % of patients with leptospirosis. Lastly, pulmonary hemorrhage was identified only in 1.4 % of patients but was seen in 25 % of leptospirosis cases.

In multinomial logistic regression analysis, leptospirosis was associated with a higher risk of adverse outcomes (adjusted Risk Ratio [aRR] = 13.67, 95 % Confidence Interval [CI] = 3.79–49.30) (Table 3). DF was associated with a lower proportion of males (aRR = 0.56, 95 % CI = 0.34–0.93) when compared to UI. Leptospirosis had a substantially increased risk of adverse outcomes (aRR = 86.83, 95 % CI =8.69–867.35) and a higher prevalence of male sex (aRR = 7.79, 95 % CI = 1.48–40.90) compared to DF.

**Table 3:** Multinomial logistic regression model examining the associations of diagnosis of infection with clinical and demographic information.

Diagnosis	Dengue fever v Unrelated/Unknown illness a (base)	Leptospirosis v Unrelated/Unknown illness a (base)	Leptospirosis v Dengue fever (base)
Adverse outcome (none)	Reference	Reference	Reference
ICU/Death	0.15 (0.02–1.27)	**13.67 (3.79–49.30)**	**86.83 (8.69–867.35)**
Age (continuous)	0.99 (0.98–1.01)	0.99 (0.96–1.03)	0.99 (0.96–1.03)
Sex			
Female	Reference	Reference	Reference
Male	**0.56 (0.34–0.93)**	**4.37 (0.86–22.10)**	**7.79 (1.48–40.90)**
Race			
White	Reference	Reference	Reference
Non-White	0.69 (0.38–1.27)	0.66 (0.16–2.79)	1.06 (0.23–4.80)
Schooling			
≤ 8 years	Reference	Reference	Reference
> 8 years	0.87 (0.52–1.45)	0.53 (0.16–1.78)	0.61 (0.17–2.13)

a Unrelated/Unknown includes 7 cases where pathogens where other etiologies were identified: 2 influenza A cases, 2 Hantavirus cases, 2 cases of advanced malignancy with associated sepsis, and 1 acute HIV infection.

## 3 Discussion

Patients in this study had a high index of suspicion for leptospirosis infection however, DENV was the most likely confirmed pathogen. Our data offers some demographic and clinical clues that may help distinguish leptospirosis from DF. UI was the most numerous patient group, highlighting the need for broader diagnostic testing in post-flood outbreaks. Two cases of Hantavirus infection, largely associated with rodent exposure, and two cases of influenza were diagnosed even though these pathogens were not being screened for in the outpatient setting. With crowded shelter conditions, respiratory pathogens may have played a significant role in the outbreak of illness. Bunyaviral, respiratory, and enteric pathogens may contribute significantly to morbidity and mortality, yet without systematic surveillance, we are limited in our ability to study their epidemic dynamics following environmental disasters.

Despite initial concerns about leptospirosis following the mass flood exposure of May 2024 in south Brazil, DENV infection predominated. This was unexpected given that the flooding event occurred during autumn, a time when temperatures typically drop below the optimal range for mosquito activity in south Brazil [22]. Further, DENV was not considered endemic to this region, where cooler temperatures limited permanent mosquito populations [23]. However, this trend has shifted over the past two decades, with the first autochthonous case of DF reported in 2007 and the first major outbreak occurring in 2016 [24,25]. Although *A. aegypti* was first recorded in the Rio Grande do Sul in 1995, its geographic and seasonal range has expanded substantially. In 2020, *A. aegypti* was recorded in over 450 municipalities compared to only 58 in 2008 [26]. Further, between 2007 and 2023, the state reported over 126,000 cases of DF and 142 deaths, with a significant increase in incidence and mortality rates between 2022 and 2023 [27].

In 2024, Brazil experienced its worst dengue outbreak in history with 6,590,575 probable DF cases, 5872 confirmed deaths, and 1136 deaths still under investigation [28]. Rio Grande do Sul was not spared from this outbreak, with 57,899 dengue cases reported between May through June 2024, compared to 18,195 cases during the same period in 2023 [29]. There is an urgent need to examine the impact of climate change on the geographic distribution of arboviral vectors, especially on the expansion of *Aedes aegypti* populations [30]. Public health efforts should prioritize ongoing vector surveillance and climate-adaptive disease prevention strategies to mitigate the growing burden of dengue in south Brazil. Finally, in situations where patients were provided with antibiotics at the time of symptom onset, treatment benefits were not readily observed, contributing to the uncertainty of antibiotic recommendations for management of leptospirosis [31].

Of note, leptospirosis is a disease that has higher prevalence in south Brazil, with a progressive increase in cases following flooding events [32]. This increase has not only been attributed to more frequent flooding but also increased health and social vulnerability in specific population groups [10]. In this study, we found that male sex prevailed among leptospirosis cases but was more evenly distributed in DF. This is consistent with most studies of leptospirosis in Brazil, where most cases occur among males [10,33]. Despite nearly universal reporting of exposure to flood water for most participants in this study, differences in sex-based occupational and post-flood activities may explain the male predominance [34]. For example, some male participants in the follow-up study reported participating in weeks-long rescue efforts, which involved wading through flood waters. This likely placed them in a protracted and continual exposure to flood waters. This may also explain why males disproportionately made up the UI category, which may have consisted of water-borne pathogens that were not screeened for. Proximity or direct contact with rats and occupational hazards were relevant but not predictive, likely due to massive exposure. Patients in this study largely came from neighborhoods in Porto Alegre that were submerged by flood waters for several weeks, providing a greater chance of exposure to various waterborne or arboviral pathogens [35]. Asking about insect bites, reliance on contaminated food or water, and lack of sanitation may have provided additional information to distinguish between infections.

This study identified some distinguishing clinical features between diagnoses. Headache was significantly more prevalent in DF, whereas respiratory symptoms, jaundice, and acute kidney injury were hallmarks of leptospirosis. Pulmonary hemorrhage was observed in over a quarter of leptospirosis cases but was absent in DF and UF cases. Pulmonary hemorrhage is a well-known and severe complication of leptospirosis and is associated with high morbidity and mortality [36]. Interestingly, calf pain and conjunctivitis often cited as important signs for leptospirosis, did not differ between groups [37].

Of note, the UI group had high rates of symptoms strongly suggestive of enteric and respiratory infections, with nearly half reporting nausea/vomiting and diarrhea, and nearly a third presenting with respiratory symptoms. This is expected as flood waters increase the risk for *E. coli*, rotavirus, cryptosporidium, and legionella infections with the disruption of farmland, sewers, and other sources [38,39]. Rio Grande do Sul has an extensive livestock industry, particularly with cattle, swine, and sheep, which further contributes to zoonotic spillover risk during flooding events. Intensive agricultural and livestock practices, combined with the increasing frequency of extreme weather events, may be creating the ideal conditions for the transmission of not only leptospirosis but also other zoonotic infections, including *Cryptosporidium* spp., antibiotic-resistant *Enterobacteriaceae*, *Salmonella* spp.*, Giardia spp*, and *Toxoplasma gondii* [40]. Given these complex environmental and epidemiological interactions, a climate-resilient surveillance system integrating veterinary, environmental, and human health data is essential for mitigating future outbreaks and improving outbreak preparedness in flood-prone regions.

Furthermore, the high rate of respiratory symptoms suggests the possibility of an undetected outbreak of respiratory infections. A study of 37 shelters following the Japanese tsunami in 2011 found that the incidence of acute respiratory illnesses increased with shelter crowding [41]. Predictably, influenza A was detected in some of our patients, which could have spread quickly in crowded shelters. Additional studies on local shelters are needed to understand respiratory pathogen transmission dynamics and improve sheltering conditions in future disasters.

Hantavirus, a rodent-borne pathogen with a high fatality rate, is endemic to south Brazil and was found to account for a subset of undiagnosed respiratory infections in the UI group [42]. The virus is primarily found in rodent urine and transmits through direct exposure or aerosolized particles [43]. As floodwaters displaced rodents from their natural habitats, they may have concentrated in shelters and homes, increasing human exposure and the likelihood of transmission. Additionally, as waters receded, large-scale cleanup efforts could have further elevated the risk, as individuals handled potentially contaminated mud and dust without adequate respiratory protection. While public health advisories recommended wearing gloves to prevent leptospirosis, hantavirus transmission through airborne particles was not widely emphasized, leaving a critical gap in preventive measures. This underscores the need to expand post-flood surveillance to include emerging pathogens and improve public health responses to environmental disasters.

The findings of this study support the need for a One Health approach to epidemic preparedness in the context of extreme weather events. One Health emphasizes the complex interaction between human, animal, and environmental health, and is particularly relevant in zoonotic epidemics driven by environmental disruption [44,45]. Future outbreak mitigation efforts should prioritize data sharing across medical, veterinary, and environmental sectors, as well as early warning systems that integrate ecological and meteorological indicators.

This study has several limitations. First, selection bias may have influenced our findings, as only patients reported to the SUS database were included. Additionally, PCR and IgM serologies may have variable sensitivity and specificity depending on the timing of sample collection. For example, we found three new cases of DENV with IgM/IgG serologies at the subsequent study visit after these patients initially tested negative. Furthermore, the interpretability of the UI category was limited as no other pathogens were screened for. The small sample size of confirmed leptospirosis necessitates caution when interpreting findings. Despite these limitations, our study demonstrated that in the event of mass urban flooding, broader surveillance is essential as leptospirosis may play a minor role as compared to other pathogens.

## 4 Conclusions

Following the significant urban flooding event of May 2024 in the state of Rio Grande do Sul, Brazil, a high concern for a leptospirosis outbreak guided public health measures. However, leptospirosis was numerically overshadowed by DF and other undetermined infections. Exposure to flood water did not predict diagnosis while symptomatology largely overlapped between groups. Although some clinical findings offered diagnostic clues, this study highlights the need for integrated communication between medicine, public health, veterinary medicine, and ecologists as well as improved diagnostic capabilities. The use of multiplex arrays to screen a broad set of potential water and vector-borne pathogens in the setting of catastrophic climatic events should be implemented to facilitate prompt diagnoses, treatment and contain outbreaks.

## CRediT authorship contribution statement

**Christopher J. Hernandez:** Writing – review & editing, Writing – original draft, Validation, Methodology, Investigation, Formal analysis, Data curation, Conceptualization. **Greici Gunzel:** Writing – review & editing, Resources, Methodology, Investigation. **Clarice Ritter:** Writing – review & editing, Supervision, Project administration, Investigation, Data curation. **Roberto Carlos Freitas Bugs:** Writing – review & editing, Supervision, Resources, Project administration, Methodology, Investigation. **Thiago Rocha:** Validation, Supervision, Resources, Project administration, Methodology, Investigation. **Trevon Fuller:** Writing – review & editing, Methodology, Investigation, Formal analysis, Data curation. **Patricia Brasil:** Writing – review & editing, Validation, Resources, Methodology, Investigation, Funding acquisition. **Ivana Rosângela dos Santos Varella:** Writing – review & editing, Supervision, Resources, Project administration, Methodology, Investigation, Funding acquisition, Formal analysis, Data curation. **Maria da Graça Pimenta Machado:** Supervision, Resources, Project administration, Methodology, Investigation, Data curation. **Carina Guedes Ramos:** Writing – review & editing, Resources, Project administration, Methodology, Investigation. **Ângela Piccoli Ziegler:** Writing – review & editing, Supervision, Resources, Project administration, Methodology, Investigation. **Breno Riegel Santos:** Writing – review & editing, Supervision, Resources, Project administration, Methodology, Investigation. **Marineide Gonçalves de Melo:** Writing – review & editing, Supervision, Resources, Project administration, Methodology, Investigation. **Karin Nielsen-Saines:** Writing – original draft, Validation, Supervision, Project administration, Methodology, Investigation, Funding acquisition, Formal analysis.

## Ethics approval

This study was approved by the Research Ethics Committee of Grupo Hospitalar Conceição (CEP-GHC), under protocol number Certificado de Apresentação de Apreciação Ética: 80682624.9.0000.5530.

## Funding

This work was supported by the Grants for Emerging Researchers/Clinicians Mentorship (G.E.R.M.) Program, administered by the Infectious Diseases Society of America (IDSA) and the HIV Medicine Association (HIVMA), as well as the South American Program in HIV Prevention Research (SAPHIR) at UCLA, through NIH/NIMH grant R25 MH087222, which provided funding for mentored infectious disease research conducted by the lead author.

## Declaration of competing interest

The authors declare the following financial interests/personal relationships which may be considered as potential competing interests:

Christopher Justin Hernandez reports financial support was provided by National Institutes of Health (NIH/NIMH grant R25 MH087222). If there are other authors, they declare that they have no known competing financial interests or personal relationships that could have appeared to influence the work reported in this paper.

## Data Availability

The data that support the findings of this study are available from the corresponding author upon reasonable request.

## Appendix A Supplementary data

Supplementary material Image 1
