# Supplementary material for: Leptospirosis? An epidemiologic investigation following the historic 2024 floods in Rio Grande do Sul, Brazil
Source: One Health. 2025 Jul 19;21:101146. doi: 10.1016/j.onehlt.2025.101146 (PMC12309501; doi:10.1016/j.onehlt.2025.101146)
Supplement: Supplementary file 1 — Supplementary material [file mmc1.docx]

Supplementary Table 1. Demographic and clinical information for patients who tested for leptospirosis, DENV, or both conditions at least once.

|  | Tested for leptospirosis  (n = 485) | Tested for DENV  (n = 303) | Tested for both  (n = 283) |
| --- | --- | --- | --- |
| Age  Median (IQR) | 38 (26, 52) | 39 (26, 52) | 40 (26, 53) |
| Sex |  |  |  |
| Male | 294 (60.6) | 181 (59.7) | 171 (60.4) |
| Female | 191 (39.4) | 122 (40.3) | 112 (39.6) |
| Race |  |  |  |
| White | 380 (78.5) | 236 (78.2) | 224 (79.2) |
| Black | 67 (13.8) | 38 (12.6) | 33 (11.7) |
| Mixed | 37 (7.7) | 28 (9.2) | 26 (9.1) |
| Education |  |  |  |
| ≤ 8 years | 217 (45.5) | 137 (45.7) | 128 (45.7) |
| > 8 years | 260 (54.5) | 163 (54.3) | 152 (54.3) |
| Healthcare |  |  |  |
| Outpatient | 419 (86.4) | 259 (85.5) | 240 (84.8) |
| Hospitalization | 66 (13.6) | 44 (14.5) | 43 (15.2) |

1. 202 among the 485 who tested for leptospirosis did not test for DENV.

20 among the 303 who tested for DENV did not test for leptospirosis.

Supplementary Figure 1. Zip code distribution of cases by diagnosis in the state of Rio Grande do Sul, Brazil.


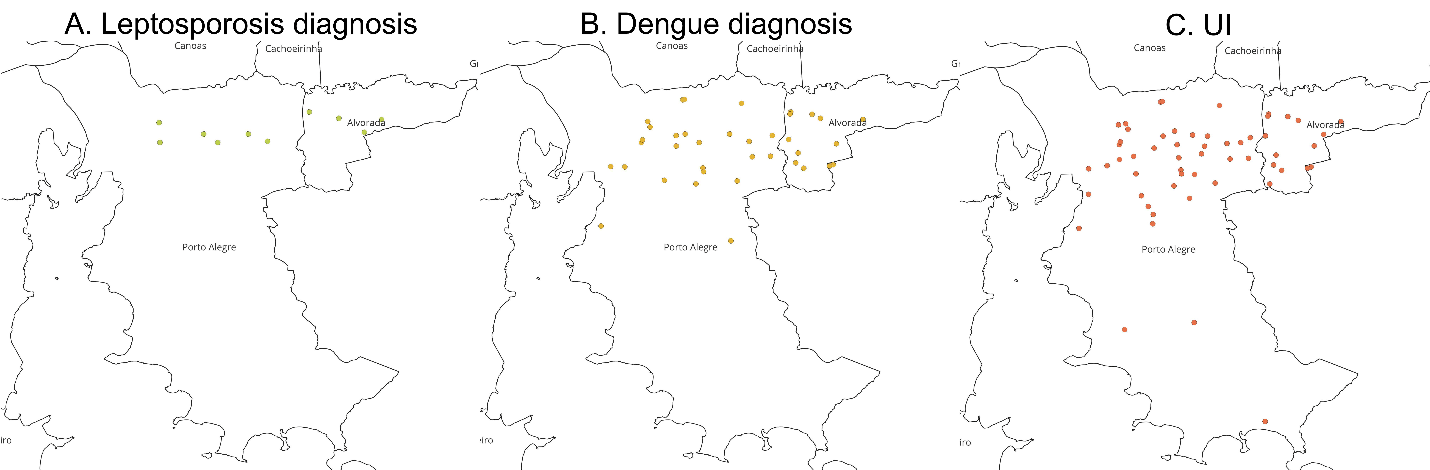


c. UI is the Unrelated/Unknown illness group.
